# Infection strategies of *Salmonella* Typhimurium for gut-lumen colonization: Overcoming host defenses, exploiting host responses, and adapting to the enteric niche

**DOI:** 10.1080/29933935.2026.2671472

**Published:** 2026-05-12

**Authors:** Tsuyoshi Miki, Masahiro Ito, Takeshi Haneda, Nobuhiko Okada, Yun-Gi Kim

**Affiliations:** a Department of Microbiology, School of Pharmacy, Kitasato University, Tokyo, Japan

**Keywords:** *Salmonella* Typhimurium, infection strategy, gut colonization

## Abstract

Diarrheal disease is a leading cause of child morbidity and mortality globally, largely resulting from contaminated food and water and exposure to enteric pathogens. *Salmonella enterica* serovar Typhimurium (*S*Tm) is an enteropathogenic bacterium that infects the gastrointestinal tract using diverse strategies that are still being elucidated. Meanwhile, the gut comprises a complex ecosystem known as the microbiome, which is densely inhabited by microbial communities. The microbiome confers colonization resistance against enteropathogenic bacteria, whereas *S*Tm can overcome these defenses to establish infection. Here, we review *S*Tm infection strategies in the gut, with a particular focus on evidence from mouse models. Understanding *S*Tm virulence mechanism and adaptation strategies may inform the development of targeted interventions to prevent and treat gastrointestinal infection.

## Introduction

Gastrointestinal infections caused by enteropathogenic bacteria are a major global health problem, with significant morbidity and mortality. The main symptom is severe diarrhea, which is a leading cause of death among infants and children, especially in developing countries. Furthermore, global prevalence of antibiotic-resistance *Enterobacteriaceae* strains represents an emerging threat for treatment of gastrointestinal infections.^1^ ^,^ ^2^ Antibiotics are often effective for treating bacterial infections by killing the pathogen; however, antibiotic therapy for many gastrointestinal infections is problematic for several reasons.^3-5^ (i) Limited benefit: Antibiotics often do not rapidly resolve diarrheal symptoms. (ii) Unintended effects: Antibiotics can disrupt the intestinal microbiota, leading to prolonged dysbiosis^6^ and a pro-inflammatory state. (iii) Risk: In some cases, e.g., in infection by enterohemorrhagic *Escherichia coli* (EHEC), antibiotic administration may increase the risk of serious complications. Therefore, alternative antimicrobial strategies beyond traditional antibiotics are needed to prevent and treat gastrointestinal infections.^7^

Enteropathogenic pathogens—including *Citrobacter rodentium* (a model for enteropathogenic *E*. *coli* [EPEC] and EHEC), *E*. *coli*, *Salmonella enterica* serovar Typhimurium (hereafter *S*Tm or *Salmonella*), *Shigella flexneri*, and *Yersinia enterocolitica*—infect the gastrointestinal tract and can colonize the gut lumen by outcompeting the microbiota.^8^ Similarly, pathobionts such as adherent-invasive *E*. *coli* (AIEC) are suspected contributors to inflammatory bowel disease (IBD), in part because they can repeatedly colonize the gut in patients. In contrast, the gut microbiome confers resistance to enteric pathogens, with a complex microbial community known as the gut microbiota protecting against pathogen colonization. This protection, referred to as colonization resistance (CR), comprises multiple defense mechanisms, including occupation of ecological niches, nutrient competition, production of antimicrobials, and modulation of mucosal immune responses.^9^ ^,^ ^10^ During the initial phase of gastrointestinal infection, CR limits pathogen colonization through niche preemption and niche modification, thereby reducing the pathogen load. Subsequently, infection-triggered inflammation and other host immune responses can further restrict pathogen growth in the gut lumen. However, virulence factors can ultimately overcome CR, enabling expansion within the gut ecosystem and, in some cases, invasion of the intestinal mucosa. At the same time, inflammation can weaken CR by disrupting the microbiota community structure, creating conditions that favor pathogen outgrowth. In this review, we discuss how *S*Tm colonizes the gut lumen by breaching mucosal defenses and adapting to the microbiome environment to overcome CR. Because *Salmonella* and *E*. *coli* are widely used models for studying host–pathogen interactions in established animal models of infection. Insights from these models may inform the development of new therapeutic strategies for gastrointestinal infection and IBD.

### *S*. *enterica* serovar Typhimurium(*S*Tm)

**Figure 1.**
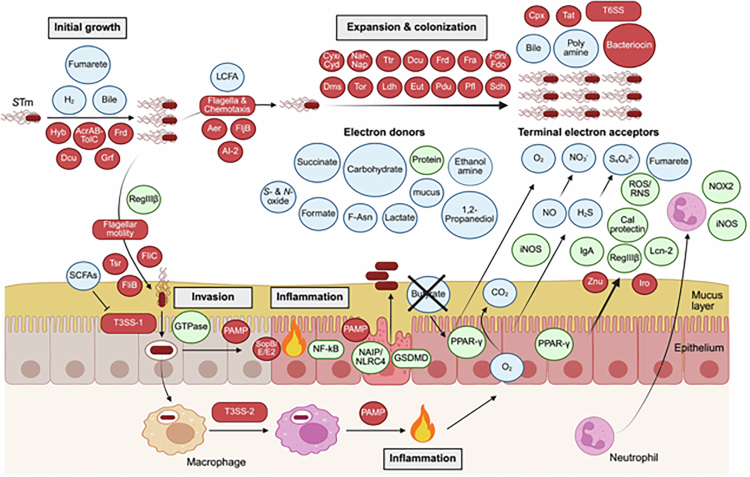
Model for gut infection with *Salmonella* Typhimurium. Upon arrival in the intestinal tract, *Salmonella* Typhimurium (*S*Tm) starts to grow and prepares to invade intestinal epithelial cells (IECs). During initial growth, *S*Tm utilizes H_2_ and fumarate and resists bile acids. A population of *S*Tm approaches the intestinal mucosa by flagellar motility to initiate invasion into IECs. The invasion depends on type 3 secretion system 1 (T3SS-1), allowing intracellular *S*Tm to induce inflammatory responses. The NAIP/NLRC4 signaling elicits epithelial contraction and expulsion. *S*Tm survives in immune cells such as macrophages in the lamina propria in a T3SS-2–dependent manner, and also elicits inflammation in a pathogen-associated molecular pattern (PAMP)–dependent manner. The inflammation causes epithelial oxygenation, enabling *S*Tm to utilize oxygen (O_2_) for aerobic respiration. On the other hand, inflammation recruits neutrophils that transmigrate across the mucus layer into the intestinal lumen and generate reactive oxygen species (ROS), resulting in production of terminal electron acceptors such as nitrate (NO_3_^−^) and tetrathionate (S_4_O_6_^2^^−^) in the gut lumen. Importantly, the resulting electron acceptors enable *S*Tm to consume limited nutrients (electron donors), such as fermentation products (carbohydrates), ethanolamine, and fructose-arginine (F-Asn) in the anaerobic environment for luminal expansion and colonization of *S*Tm. Red circles indicate *S*Tm-derived factors, whereas blue and green ones represent host-derived molecules and proteins, respectively. Abbreviations: SCFAs, short chain fatty acids; LCFA, long chain fatty acid; NAIP/NLRC4, NLR family apoptosis inhibitory protein/NLR CARD-domain containing protein 4; GSDMD, gasdermin D; PPAR-*γ*, peroxisome proliferator-activated receptor gamma; Lcn-2, lipocalin 2; RegIIIβ, regenerating islet-derived protein III beta; RNS, reactive nitrogen species; NOX2, NADPH oxidase 2; iNOS, inducible nitric oxide synthase. Created with BioRender.com.

*S*Tm is a major cause of diarrheal disease globally.^2^ ^,^ ^11-13^ In most healthy individuals, *S*Tm infection is self-limiting; however, in infants and immunocompromised individuals, it can progress to bloodstream infection and disseminate to systemic sites, leading to life-threatening disease such as sepsis and, in severe cases, death.^13-15^ Unfortunately, a vaccine to prevent *S*Tm-associated diarrhea is not currently available, and antibiotic therapies have been shown to be ineffective.^4^ Thus, understanding *S*Tm infection mechanisms may be the best way to develop new anti-infective agents to protect high-risk groups (Figure 1).

The human *S*Tm infection, often caused by contaminated food or water, leads to inflammatory disorders ranging from gastroenteritis to enterocolitis.^16^ Common symptoms include headache, nausea, vomiting, abdominal pain, myalgia, and non-bloody diarrhea, which begin 6–48 hours after ingestion. Since the infection is non-systemic, *S*Tm-infected patients exhibit less splenomegaly and hepatomegaly.^17^ The gastrointestinal salmonellosis is usually self-limiting, lasting from a few days to 10 d.^18^ However, bacteremia and focal systemic infections occur in susceptible individuals, such as infants, young children, and immunocompromised patients.^19^ Thus, risk factors for gastrointestinal salmonellosis represent age (the very young and the elderly), dysbiosis, diabetes, malignancy, rheumatological disorders, reticuloendothelial blockade, HIV infection, and immunosuppression treatment.^17^ Antibiotic therapy is not endorsed for treatment of *S*Tm gastroenteritis in healthy individuals, whereas patients with risk factors for extraintestinal spread of infection and who are severely ill should be treated with antibiotics.

Oral ingestion of contaminated food and water mainly triggers the gastrointestinal infections with *S*Tm in humans and other species.^20^ ^,^ ^21^ After ingestion, *S*Tm must pass through the stomach containing acidic gastric contents to colonize the digestive tract. Thus, acid adaptation is associated with the virulence of *S*Tm, leading to survival in the stomach. The gastrointestinal tract is the primary site for acute *S*Tm infection, where *S*Tm binds to intestinal epithelial cells (IECs) using adhesins, resulting in membrane ruffling and bacterial internalization into the host cell cytoplasm.^22-24^ *S*Tm within host cells can replicate by forming the *Salmonella*-induced filaments.^25^ Intracellular replication confers protection from host immune responses, and gut infection with *S*Tm has been established. In contrast, by recognizing the pathogen insult, the host launches immune responses, including the recruitment of immune cells like neutrophils and macrophages at the site of infection.^26^ These immune cells engulf *S*Tm and then try to eliminate it. Importantly, at the same time, the cells produce proinflammatory cytokines. However, *S*Tm escapes from immune attack by such as inhibiting phagolysosome fusion.^27^ Finally, *S*Tm successfully invades the mucosal barrier and, in the case of immunocompromised hosts, spreads towards systemic sites, causing systemic infection.^26^ ^,^ ^28^

The virulence strategies of *S*Tm, including cell invasion, induction of inflammation, and intracellular replication, are primarily driven by the type-three secretion system (T3SS). This system encodes a needle-like injection structure that directly delivers bacterial virulence factors, known as effectors, into the host cell cytosol.^29^ *S*Tm notably contains around 50 effectors, each playing distinct roles such as facilitating host cell invasion, promoting inflammation, aiding systemic spread, contributing to intracellular replication, exerting anti-inflammatory effects, inducing the host cell death, and modulating virulence.^30^ In addition to T3SS, *S*Tm’s survival within host tissues and cells is crucially dependent on its ability to withstand host-derived antimicrobial responses. Macrophages and neutrophils attempt to eliminate *S*Tm by producing reactive oxygen species (ROS) and reactive nitrogen species (RNS).^31^ ^,^ ^32^ To successfully establish infection, it is notable that *S*Tm must encounter host oxidative stresses by detecting environmental ROS through redox sensors like SoxR/S and evading these defenses.^33^ ^,^ ^34^ Immune defects significantly increase the risk of developing bacteremia in *S*Tm gastrointestinal infections, implying that typhoidal *Salmonella* serovars, such as Typhi and Paratyphi A, have unique mechanisms to evade the ROS attack from phagocytes like macrophages and neutrophils. Specifically, while *S*Tm infection triggers strong ROS production in human neutrophils, this response is notably reduced in infections with *S*. Typhi or *S*. Paratyphi A.^35^ Furthermore, for intracellular survival, *S*Tm creates a favorable immune environment by regulating autophagy and programmed cell death pathways, including apoptosis, pyroptosis, and necroptosis, to effectively suppress inflammation, delay cell death, and acquire necessary resources.^36^ Besides the virulence strategies of interaction with host cells, *S*Tm movement by flagellar-based motility also contributes to the pathogenesis of this pathogen.^37-39^

Environmental adaptation linked to virulence regulation is crucial for *S*Tm infection. For instance, two-component signal transduction systems (TCSs) like PhoPQ and CpxRA serve as vital pathways in *S*Tm pathogenesis.^40-43^ TCS, comprising the histidine kinase sensor and the cytoplasmic response regulator, is particularly critical in managing the envelope stress response.^44-46^ The resulting alteration of gene expression profiles allows *S*Tm to thrive in a wide variety of niches. Earlier work has shown that 86% of all *S*Tm genes are expressed in at least one environment.^47^ Virulence determinants such as adhesin, T3SS, and flagella are transcriptionally regulated in response to environmental cues, for instance the stomach acidity or intestinal hypoxia. Therefore, the adaptability and versatility, characterized by complex regulatory mechanisms and significant metabolic capacity, are vital for *S*Tm pathogenesis.

Gut inflammation triggers a bloom of *S*Tm in the gut lumen, creating a favorable niche for its growth.^48^ This inflammation leads to significant ecological changes, including the secretion of antimicrobial proteins that preferentially inhibit luminal growth of commensals,^49^ ^,^ ^50^ the production of high-energy nutrients for *S*Tm,^38^ and the formation of alternative electron acceptors that fuel its growth.^51^ At the end of the acute infection, a protective secretory immunoglobulin A (sIgA) antibody response aids in remission and the regrowth of normal microbiota.^52^

Very high densities (>10^9^ bacteria/gram of intestinal content) of *S*Tm in a favorable niche lead to within-host evolution of the pathogen, including acquisition of new genes via horizontal gene transfer (HGT) and accumulation of gene mutations, which mainly impact the transmission of virulence.^53^ ^,^ ^54^ Conjugative transfer of plasmids occurs between *S*Tm and *E*. *coli* even in the absence of gut inflammation.^53^ Meanwhile, gut inflammation contributes to temperate bacteriophage transfer between strains of *S*Tm, in which the SOS response controls the expression of the lytic cycle genes of the phage.^54^ On the other hand, it is notable that within-host evolution of *S*Tm results in the evolution of avirulent mutants with a defect in a central positive regulator of T3SS gene expression.^55^ The *S*Tm mutant has lost the functional T3SS, whereas the luminal growth rate of the mutant is faster than the strain expressing T3SS.^55^ ^,^ ^56^ Furthermore, the mutant that cannot enter the host cell also escapes from intracellular killing in the inflamed gut tissue.^55^ ^,^ ^57^ Finally, the rise of mutations in the within-host evolution leads to disadvantages for long-term colonization of *S*Tm since the inflammation cannot be sustained by the accumulation of mutants, incapable of eliciting host inflammation. In addition to within-host evolution, high densities of *S*Tm are also associated with transmission to the next host, whereas the transmission of mutants prevents disease. Importantly, antibiotic treatment results in detrimental consequences for transmission-mediated disease. Antibiotics kill *S*Tm cells in the gut lumen; however, *S*Tm cells expressing T3SS survive in the gut tissue and can reseed the lumen upon antibiotic withdrawal, leading to successful transmission of the virulence to the next host.^58^

*S*Tm is a zoonotic pathogen with a broad host range, capable of colonizing the gastrointestinal tract of both humans and animals. Notably, multiple host-adapted *S*Tm variants have emerged, with pathovariants breaching the intestinal mucosal barrier and disseminating to systemic sites, causing severe invasive disease. ST313, a remarkable example of these invasive variants, causes bloodstream infections and has adapted to humans, particularly in sub-Saharan Africa.^59-61^ This adaptation involves genome degradation with mutations in genes related to oxidative stress resistance, biofilm formation, carbon metabolism, and gastrointestinal pathogenesis.^62^ Notably, inactivation or pseudogenization of genes involved in flagellar motility, lipid A modification, and T3SS effectors suggests that loss of the capacity for colonizing the gut lumen may be linked to evolutionary host adaptation of *S*Tm.^62^ Additionally, it is suggested that antibiotic use for *S*Tm infections in sub-Saharan Africa may have influenced ST313’s evolution.^62^ ^,^ ^63^ Furthermore, comparative analyzes of evolution between *S*Tm and typhoidal *Salmonella* serovars show that maintaining genomic stability helps *S*Tm to function metabolically in the gut. For instance, the retention of the *ydiQRSTD* operon, which encodes an anaerobic *β*-oxidation of butyrate, provides metabolic adaptation benefits to *S*Tm during the gastrointestinal infection.^64^ As another example, the *fljBA-hin* locus, a genetic element acquired during the evolution of *S*Tm, enables the bacterium to express two types of flagellin proteins, FliC (phase 1) and FljB (phase 2), which are crucial for its motility and virulence.^65^ These proteins form the structural subunits of the flagellum, a whip-like appendage that aids in bacterial movement. However, due a genetic mechanism called phase variation, regulated by the invertible element *hin*, these two flagellin genes cannot be expressed at the same time.^66^ As a result, *Salmonella* strains can be classified as biphasic, monophasic, or non-motile, depending on whether they express both, one, or neither of the flagellar phases. Notably, certain highly invasive *Salmonella* serovars, such as Typhi, Paratyphi A, and Choleraesuis, are monophasic, express *fliC* gene.^67^ The loss of the *fljB* gene in these strains remains unclear, but previous studies suggest that the integration of the Tn21 transposon into the *fljBA-hin* locus provides resistance to heavy metals and antibiotics, aiding in the bacterium’s survival and spread, especially in environments associated with food production.^68^ ^,^ ^69^ Conversely, pandemic monophasic variants of *S*Tm found in Vietnam have been linked to invasive nontyphoid *Salmonella* infections in HIV-infected patients, as they have reacquired the phase 2 flagellum, highlighting the potential role of diphasic flagellin expression in the pathogenicity of *S*Tm.^70^

The gastrointestinal tract, which *S*Tm preferentially infects, is equipped with multiple layers of protection against *S*Tm infection.^71^ These protective layers consist of the complex microbiota in the gut lumen, the epithelial cell layer with mucus, and immune cells in the lamina propria.^9^ ^,^ ^72^ ^,^ ^73^ In contrast, *S*Tm can colonize the gut through its sophisticated pathogenic strategies. To summarize, *S*Tm initiates dynamic and previously unrecognized interactions with the microbiota, the intestinal mucosa, and the mucosal immune system. Multiple *in vitro* models, such as those using cultured cells and organoids, are currently available to investigate the interplay. However, these models may be insufficient to recapitulate the complexity of dynamic interactions. Therefore, by focusing on accumulated evidence from mouse models, we present an in-depth discussion on how *S*Tm infects the gastrointestinal tract, particularly the gut colonization below. Thus, we hope that understanding the gastrointestinal tract colonization by *S*Tm could lead to the development of alternative therapeutic interventions for its infection.

## *S*Tm initial luminal growth

In the initial phase of infection, *S*Tm seeks mainly to invade IECs, which is also linked to dissemination into deeper tissues. Thus, upon arrival in the large intestine, *S*Tm starts to grow in the gut lumen, because even low numbers of enteropathogenic bacteria can cause gastrointestinal infection. The microbiota is intact and thereby contributes to the initial growth of *S*Tm. *S*Tm exploits molecular hydrogen (H_2_), an intermediate of anaerobic microbiota metabolism, to perform anaerobic respiration (fumarate respiration).^74^ H_2_ acts as an electron donor for *hyb* hydrogenase,^74^ ^,^ ^75^ and the electrons are transferred to fumarate reductase (Frd), which uses fumarate as the terminal electron acceptor, thereby reducing fumarate to succinate.^74^ Fumarate is generated from diet- and/or microbiota-derived L-aspartate and/or L-malate after uptake by DcuABC.^76^ Furthermore, a recent study has shown that uptake of the Amadori compound fructoselysine via the Gfr system plays a context-dependent role in *S*Tm initial growth in the microbiota-colonized gut.^77^ Similarly, dietary shifts can influence context-dependent initial growth of *S*Tm. High-fat-diet-induced bile acid production facilitates *S*Tm gut colonization in a context-dependent manner, and this effect requires AcrAB/TolC-dependent bile resistance; however, competitive *E*. *coli* can protect against *S*Tm colonization under high-fat-diet conditions.^78^

As mentioned above, *S*Tm grows to high densities and exceeds a certain threshold. Once a critical density is reached, *S*Tm deploys two major virulence determinants: the *Salmonella* pathogenicity island 1 (SPI-1)-encoded type-three secretion system (T3SS-1), which enables invasion of non-phagocytic cells (e.g., IECs) and induces pro-inflammatory cytokines,^79-81^ and the SPI-2-encoded T3SS-2, which promotes intracellular survival and replication within host cells.^81-83^ Thus, the invasive phenotype into the gut tissue is associated with disease status such as diarrhea and gut inflammation.^81^ ^,^ ^84^ ^,^ ^85^ Environmental cues in the gut lumen, such as osmolarity, pH, oxygen tension, bile acids, Mg^2+^ concentration, and short-chain fatty acids (SCFAs), regulate *S*Tm invasion by influencing the expression of T3SS-1 genes.^86-90^ Notably, since the concentrations of SCFAs vary throughout the gastrointestinal tract,^91-93^ in the small intestine with low total SCFAs, a predominant acetate induces invasion, whereas high total SCFAs in the colon, and greater concentrations of propionate and butyrate suppress the expression of T3SS-1 genes.^89^ ^,^ ^90^ Consistent with these reports from *in vitro* studies, the SCFAs propionate and butyrate play a protective role in *Salmonella* pathogenesis.^94^ ^,^ ^95^ However, a recent work combining mathematical modeling with time-resolved single-cell experiments provided unprecedented insights into how SCFAs impact single-cell behaviors of *S*Tm.^96^ In the *S*Tm-infected gut, virulence gene expression shows bistability^57^; namely, two discrete populations arise: T3SS-1–expressing cells and –nonexpressing cells, the latter of which can grow rapidly.^55^ ^,^ ^56^ ^,^ ^97^ These *S*Tm cells cooperate with each other to facilitate the gut colonization.^55^ A recent study revealed that SCFAs decreased the size of the T3SS-1-positive population by slowing the growth rate of T3SS-1–expressing cells without reducing the transcriptional activity of T3SS-1 genes.^96^ In addition to T3SS-1 expression, flagella and specific adhesins are required the approach and attachment of *S*Tm to IECs. Flagellar motility allows *S*Tm to swim to IECs and attach to the cell surface in the pre-invasion stage. A mucus layer overlays the IECs, which is mainly composed of Muc2 and contains IgA and antimicrobial proteins.^98^ IgA causes bacterial aggregation by direct interaction, leading to slower bacterial mobility in the mucus.^99^ Bactericidal lectin RegIIIγ (regenerating gene family protein III-gamma) in mice, a counterpart of human REG3A, eliminates Gram-positive commensal bacteria from the mucus layer by direct killing.^100^ In contrast, the murine homolog RegIIIβ (RegIII-beta) can bind and display a bactericidal effect on Gram-negative bacteria, and thus RegIIIβ is thought to have a similar effect on Gram-negative commensal bacteria, as evidenced by the finding that RegIIIβ is bactericidal to *Bacteroides* spp., a major group of Gram-negative commensal bacteria.^49^ ^,^ ^101^ ^,^ ^102^ Surprisingly, even though RegIIIβ can bind to *S*Tm, the pathogen is resistant to the RegIIIβ-mediated bactericidal effect; furthermore, the binding increases the flagellar-based locomotion speed of *S*Tm, resulting in increased invasion into non-phagocytic cells.^103^ Thus, *S*Tm can traverse the mucus layer efficiently through flagellar motility.^104^

## *S*Tm adherence and docking for IEC invasion

Upon arrival at the surface of IECs, *S*Tm adheres to the cells to initiate invasion. Adhesins, including fimbriae, are well known for their role in bacterial adherence to the host cell surface,^105^ ^,^ ^106^ and the *S*Tm genome encodes 21 potential adhesins.^107^ Several fimbria and T1SS-secreted adhesin SiiE have been shown to contribute to gut infection in murine models.^108^ ^,^ ^109^ However, a comprehensive understanding of the underlying mechanism remains elusive. In addition to adhesins, T3SS-1 translocases facilitate the intimate association of *S*Tm with host cells; this is known as “docking” and is a prerequisite for efficient invasion.^110^ ^,^ ^111^ On the other hand, the flagellin phase contributes to the subsequent productive invasion by targeting the invasion site during near-surface swimming of *S*Tm.^39^ ^,^ ^112^ ^,^ ^113^ FliC-expressing *S*Tm tends to remain on the cell surface, resulting in higher frequencies of invasion into host cells.^112^ Furthermore, FliB-mediated methylation of FliC flagellin enhances invasion efficiency by facilitating attachment to the cell surface, as evidenced by findings suggesting that increased surfaced hydrophobicity of the flagellar filament through methylation may improve hydrophobic interaction to host cells.^114^ The cell length of *S*Tm is also implicated in attachment efficiency to host cells. Elongated cells, possibly undergoing division, show enhanced adhesion to host cells, which may be due to their larger surface area for *S*Tm–host cell interaction.^115^

## *S*Tm invasion into IECs

After completion of the docking, entry of *S*Tm into IECs is initiated by delivery of T3SS-1 effectors into the host cells. The molecular basis of T3SS-1-dependent IEC invasion has been reviewed elsewhere.^79^ ^,^ ^116^ Three key effectors of T3SS-1, SopB, SopE and SopE2 (SopB/E/E2), enable the coincident internalization of several bacteria into cell lines by triggering membrane ruffling resulting from activation of Rho-GTPases, Arf-GTPases, and formins.^117-120^ The T3SS-1 effectors SipA and SipC also contribute to membrane ruffling by promoting actin nucleation and bundling.^121-123^ Interestingly, SipA plays a distinct role in invasion, as it can mediate SopB/E/E2-independent invasion into cultured cells.^124^ ^,^ ^125^ Furthermore, SipA drives discreet invasion into the absorptive gut epithelium of mice, which appears to be distinct from SopB/E/E2-dependent invasion.^126^ Although there is accumulating evidence for the T3SS-1-dependent invasion, it is not fully understood how *S*Tm invades IECs. The individual effectors play redundant roles in *S*Tm invasion. In addition, some invasins, such as PagN and Rck, have been shown to contribute to *S*Tm invasion, independently of T3SS-1.^127-129^ Most importantly, invasion of *S*Tm relies on cellular conditions, including the cellular environment, polarity, and maturation. For example, a recent study showed that IEC differentiation toward an enterocyte/colonocyte state inhibits *S*Tm invasion compared with that in immature IECs.^130^ In addition to T3SS-1, T3SS-2 also contributes to induction of gut inflammation.^81^ ^,^ ^131^ ^,^ ^132^ T3SS-2 is involved in *S*Tm-induced epithelial barrier breakdown at later stages of infection, thereby exacerbating enteropathy in the gut.^133^ Earlier *in vitro* analyzes revealed that, in addition to the T3SS effectors, flagellum-mediated motility contributes to *S*Tm invasion of epithelial cells.^134^ ^,^ ^135^ Further research using a mouse colitis model has shown that flagellum-mediated motility and chemotaxis, driven by the methyl-accepting chemotaxis protein (MCP) Tsr, enable *S*Tm to migrate toward IECs by sensing host-derived nitrate, thereby contributing to T3SS-1–independent invasion of Peyer’s patches.^136^ On the other hand, the host glycome influences *S*Tm invasion into IECs, as evidenced by earlier work showing that *S*Tm chitinases modulate the surface glycome of IECs, thereby contributing to *S*Tm invasion of murine ileal epithelial cells.^137^

## *S*Tm-induced inflammation of IECs

*S*Tm invading IECs stimulates inflammatory responses by at least two distinct mechanisms: injection of T3SS-1 effectors that directly interfere with multiple host immune responses^81^ and pathogen-associated molecular pattern (PAMP)-induced innate immunity in IECs.^138^ ^,^ ^139^ A battery of T3SS-1 effectors, including SopB/E/E2, SopA, and SopD, are involved in the induction of inflammatory signaling: SopB/E/E2 induces inflammatory signaling by activating nuclear factor-κB (NF-κB); SopA does so via the retinoic acid-inducible gene I protein (RIG-1)/melanoma differentiation-associated protein 5 (MDA5) signaling pathway; and SopD targets Rab8 GTPase-activating protein.^140^ Perturbation of cellular responses by the proinflammatory effectors leads to the production of proinflammatory cytokines, such as interleukin-8 (IL-8), tumor necrosis factor (TNF) and IL-1β. On the other hand, pattern recognition receptors (PRRs) sense mucosal intrusions. Epithelial cell membrane-bound Toll-like receptors^138^ and Nod-like receptors (NLRs) forming inflammasomes in the cytosol^141^ recognize the pathogens, resulting in the production of proinflammatory cytokines. The proinflammatory cytokines lead to the recruitment of inflammatory cells, including macrophages and neutrophils, to the gut lumen. Most importantly, the cell repositioning changes the intestinal lumen environment and thereby dampens CR activity and allows the competitive colonization of *S*Tm. Additionally, the NAIP/NLRC4 inflammasome-mediated recognition elicits the death and expulsion of the *S*Tm-infected IECs into the gut lumen to restrict the pathogen’s proliferation, independently of the presence of proinflammatory cytokines such as IL-18 and IL-1β.^142^ ^,^ ^143^ Notably, the NAIP/NLRC4 signaling elicits the epithelial contractions that may reduce the destructive effects of the subsequent IEC death and expulsion through an increase in IEC packing at the infectious site.^144^ Thus, NAIP/NLRC4 inflammasome dynamics that are tailored to development stages and the NAIP ligand concentration profoundly affect the outcome of *S*Tm gut infection.^145-148^ In studies using the NAIP/NLRC4 ligands or *S*Tm bacterial cells, gasdermin D (GSDMD), which has the capacity to form membrane pores and to induce pyroptosis and secretion of pro-inflammatory cytokines, affects the IEC extrusion process.^144^ ^,^ ^149^ Since caspase-1 participates in the cleavage of GSDMD, both the innate effectors play an important role in NAIP/NLRC4-mediated protection of the intestinal mucosa against *S*Tm.^150^

Intracellular *S*Tm resides within a modified phagosome called the *Salmonella*-containing vacuole (SCV), within which the pathogen can survive and replicate.^151^ ^,^ ^152^ The T3SS-1 effectors SopB/E/E2, SipA, and SopF influence the maintenance of SCV, and thereby contribute to survival and localization within IECs.^153-158^ In addition to the SCV, other endomembrane compartments known as the infection-associated macropinosomes (IAMs) are formed by *S*Tm IEC infection.^159^ The SCV fuses with the IAMs, and goes through subsequent maturation steps, resulting in the formation of a large compartment within which *S*Tm can begin to replicate.^160^ ^,^ ^161^ Alternatively, a small population of *S*Tm in SCV can escape into the host cell cytosol as a secondary niche, in which the pathogen is eliminated by xenophagy or replicates at very high rates.^142^ ^,^ ^162-165^ The hyper-replicated *S*Tm may induce NAIP/NLRC4 signaling via recognition of cytosolic lipopolysaccharide (LPS), flagellin, and T3SS component proteins, resulting in IEC pyroptosis and extrusion from the epithelial layer.^139^ It is reasonable to assume that the cytosolic *S*Tm will spread to the gut lumen by cell death and NAIP/NLRC4-mediated extrusion. Thus, SCV dynamics, including enlargement and vacuolar rupture, tune the niche of intracellular *S*Tm, affecting pathogenesis during the post-invasion phase.^161^ Furthermore, the cytosolic *S*Tm and ruptured SCV are targeted by autophagy in selective manners.^166-168^ Upon damage of the SCV, *S*Tm is exposed to the cytosol, initiating xenophagy that restricts the intracellular growth.^169^ By contrast, to escape from xenophagy, successful pathogens can prevent the initiation of autophagy. *Shigella* secreting IcsB has been shown to interfere with tracking pathways of autophagy.^170^ In the context of *S*Tm, the pathogen inhibits activation of the AMP-activated protein kinase-dependent pathway of mTOR, and thereby inhibits initiation of autophagy.^171^ In other ways, the T3SS effectors SopF and SseF/SseG are involved in antagonism to autophagy-mediated pathogen elimination by disrupting the V-ATPase-ATG16L1 association and Rab1A signaling respectively.^158^ ^,^ ^172^ On the other hand, autophagy repairs the SCV membrane damage harboring T3SS-1-expressing *S*Tm at the early stages of infection, which is required for SCV maturation and promotion of T3SS-2 expression.^173^ In addition to the effects on intracellular pathogens, autophagy contributes to the mucosal barrier of the intestine by influencing the extracellular environment. It is well known that mutations in autophagy genes attenuate the mucosal barrier, at least in part due to gut dysbiosis and dysfunction of epithelial cells such as goblet and Paneth cells.^174^ ^,^ ^175^

Upon arrival in the intestinal lamina propria via T3SS-2–dependent traversal,^176^ *S*Tm encounters phagocytes, including neutrophils and monocytes, which migrate in response to inflammatory signals to target the pathogen. Neutrophils protect the host by reactive oxygen species (ROS)-mediated killing of *S*Tm.^177^ In addition, engulfment of *S*Tm by neutrophils triggers inflammasome activation, leading to degranulation and IL-1β secretion^178^ or NETosis.^179^ Similarly, neutrophils transmigrate into the gut lumen and reduce the luminal *S*Tm burden. On the other hand, macrophages can sample the luminal *S*Tm by extending dendrites, and engulf the pathogen in the lamina propria. In general, intestinal macrophages that reside in the lamina propria exhibit an anti-inflammatory phenotype driven by microbiota-mediated recruitment and metabolites.^180^ Furthermore, intestinal macrophages contribute to adaptive immunity by initiating an IgA response during *S*Tm infection.^181^ In the context of infection, *S*Tm invasion into the gut shifts macrophage responses from an anti-inflammatory to a pro-inflammatory phenotype by triggering the recruitment of pro-inflammatory monocytes that contribute to *S*Tm clearance via ROS production.^177^

In contrast to the protective effects, macrophages can be exploited by *S*Tm for replication within the lamina propria. The SCV in macrophages supports *S*Tm replication rather than NAIP/NLRC4-mediated pyroptosis, which is limited by downregulated expression of T3SS-1 and flagellin in the lamina propria.^148^ By contrast, replication of *S*Tm in the SCV relies on T3SS-2, which triggers of innate immune responses within host immune cells such as dendritic cells and macrophages.^182^ Furthermore, a recent report revealed that the T3SS-2 effectors SopD2 and GtgE together are required for T3SS-2–dependent gut pathology and inflammation in a mouse colitis model, although the underlying mechanism remains unclear.^183^ On the other hand, macrophages can lyse the SCV by activating the expression of guanylate-binding proteins (GBPs), resulting in release of *S*Tm and LPS into the cytosol.^184^ ^,^ ^185^ Afterwards, caspase-11 senses the cytosolic LPS and cleaves inactive GSDMD, triggering pyroptosis that leads to IL-1β secretion.^186^ Pyroptosis captures *S*Tm in pore-induced intracellular traps (PITs), promoting pathogen killing via efferocytosis.^187^ Similar to macrophages, dendritic cells (DCs) in the lamina propria can capture luminal *S*Tm by promoting dendrite extension.^188^ ^,^ ^189^ DCs containing *S*Tm migrate to the mesenteric lymph node (mLN), which is essential for initiating adaptive immune responses, whereas these cells can also permit systemic dissemination of the pathogen.^190^ Importantly, presentation of *S*Tm peptides on MHC-1 molecules via degradation in acidic phagosome and cross-presentation fosters *S*Tm-specific CD8^+^ T cell responses that limit pathogen loads at systemic sites.^191-193^ We discuss below how *S*Tm can expand in the gut lumen through the exploitation of host factors, escape from host immune responses, and adaptation to the intestinal environment.

## *S*Tm luminal expansion and colonization

Rolf Freter’s nutrient niche theory predicts that enteropathogenic bacteria can colonize the gut lumen upon finding a comfortable niche, allowing them to grow competitively.^194^ ^,^ ^195^ Gut luminal growth in the inflamed gut is a fundamental aspect of the gastrointestinal infection of *S*Tm, enabling disease and transmission, and requires *S*Tm to overcome the CR along and outcompete the commensal microbiota. In contrast, the gut microbiota can interfere with luminal growth of *S*Tm in mainly two competitive ways: nutrient competition and interference competition. Therefore, the gut colonization of *S*Tm proceeds in several phases and ways. However, it is not fully understood how *S*Tm performs the key infectious step. The intestinal ecosystem invaded by *S*Tm comprises the microbiota, mucosal immunity, and the enteropathogen *S*Tm, which interact with each other. Furthermore, the ecosystem is complicated by food, its digestion products and the intestinal mucosa. Thus, to explore the specific mechanisms employed by *S*Tm to colonize the gut lumen, mouse infection models are needed, which recapitulate the complexity of interactions in the ecosystem.^196^ ^,^ ^197^ Accumulating data resulted from mouse infection experiments have revealed that the ability to trigger and exploit intestinal inflammation plays a central role in the colonization of pathogens, including *S*Tm, in the inflamed gut.^48^ A hallmark of the intestinal inflammation caused by *S*Tm infection is release of intestinal nutrients, specific terminal electron acceptors, and antimicrobial proteins, along with oxidative species such as ROS and reactive nitric species (RNS).^198-200^ The changes in the gut-luminal milieu have a negative impact on the microbiota. Surprisingly, *S*Tm benefits from the changes by adapting and evolving metabolic and defense mechanisms to exploit the gut environment, allowing for a competitive advantage in luminal growth.^47^ ^,^ ^48^ ^,^ ^201^ Earlier work has shown that approximate 90% of all genes in *S*Tm are expressed under *in vitro* culture conditions, indicating that alterations to the gene expression profile of *S*Tm impact the adaptation and evolution to environmental perturbations such as intestinal inflammation.^47^ We will discuss the underlying mechanism by which *S*Tm can expand in the inflamed gut below.

### Flagella and chemotaxis

Flagella are the first reported factor affecting the luminal colonization of *S*Tm in the inflamed gut.^37^ In addition to a role in bacterial movement, flagella are needed for efficient colonization and induction of colitis, which is mainly due to chemotaxis, whereas recognition of flagellar subunits by innate immune receptors may be less important.^37^ Flagellar motility allows *S*Tm to access mucin-related nutrients, namely galactose-containing glyco-conjugates, which the pathogen utilizes for enhanced growth in the inflamed gut but not in the uninflamed gut.^38^ Aer mediates energy taxis towards favorable spatial niches containing tetrathionate in the inflamed gut, whereas Tsr-mediated energy taxis is nitrate respiration-dependent^202^ (see below for discussion). Both energy taxis responses contribute to the fitness advantage in the inflamed gut.

*S*Tm can express two antigenically distinct flagellins, FliC and FljB, by flagellar phase variation. Interestingly, the flagellar phase variation influences the luminal infection capacity of *S*Tm, as evidenced by the results that FliC-expressing *S*Tm cells outcompete FljB-expressing bacteria in the intestinal tract.^112^ FliC-flagella contribute to an advantageous cell invasion, resulting from increased dwelling time on cell surfaces and thereby facilitating T3SS-1–dependent cell invasion.^112^ By contrast, *fljB* expression is involved in the luminal colonization of *S*Tm.^203^ Long-chain-fatty-acid (LCFA) homeostasis maintains proper flagellar motility, as evidenced by the results that *S*Tm strain with deletion of the *fadR* gene encoding the LCFA metabolism-associated transcriptional regulator is impaired in the gut colonization, which are attributable to altered swimming behavior characterized by less frequently smooth swimming.^203^ Together, the flagellar phase variation is a critical factor influencing the abilities of *S*Tm to invade host cells and colonize the gut lumen.^112^ ^,^ ^203^ Furthermore, LCFA levels in the intestinal tract significantly influence the luminal colonization of *S*Tm by altering balance of the flagellar phase variation.^203^ The *fljB* acquisition during evolution results in enhancement of the luminal gut colonization by *S*Tm in concert with LCFA.

By chemotaxis system, *S*Tm navigates the environmental gradients of various compounds, including nutrients for luminal growth and colonization. A recent study has shown that chemotaxis towards autoinducer-2 (AI-2) of *E*. *coli* confers nutrient competition with *S*Tm.^204^ Thus, it is notable that cooperation of chemotaxis and quorum sensing plays a role in metabolic competition among Enterobacteriaceae in the gut lumen.

### Nutrient utilization

As Rolf Freter’s nutrient niche theory was discussed above, the notion of spatial competition and the heterogenous environment was incorporated to this theory, yielding an expanded theory called the Restaurant hypothesis.^205^ In the gut lumen, commensal bacteria have developed to utilize distinct nutrients for their luminal growth and share nutrients for co-existence, which include carbohydrates, amino acids, minerals, and vitamins.^206^ ^,^ ^207^ In homeostasis, nutrients are depleted by the microbiota’s utilization, and thus their availability is limited.^208^ ^,^ ^209^ Diet promotes the growth of bacteria that predominantly use the ingested diet, shaping the microbiome.^208^ ^,^ ^209^ Inflammatory responses or antibiotic treatment impact the life of the microbiota, both by altering its composition and causing the release of intracellular nutrients. Thus, importantly, the nutrient availability is shifted from the microbiota to the pathogens, including *S*Tm.^201^ ^,^ ^210-212^ This is evidenced by prior studies showing that disruption of the microbiota community by antibiotic treatment promotes the luminal growth of *S*Tm in the murine cecum.^80^ ^,^ ^213^ Thus, *S*Tm needs to obtain at least one nutrient to proliferate in the gut lumen. On the other hand, bacteria specific to the same niche share similar catabolic profiles, which play pivotal roles in competitive intestinal colonization of *S*Tm.^214^

### Carbohydrates

Nutrient metabolism, including carbohydrate fermentation, is the cardinal metabolic mode used by most members of the microbiota and is required for bacterial growth *in vivo*.^215^ ^,^ ^216^ Carbohydrates (monosaccharides) are degraded to phosphoenolpyruvate via glycolytic pathways—including the Embden-Meyerhof-Parnas pathway, the Entner–Doudoroff pathway, and the pentose phosphate pathway—which then generate ATP through substrate-level phosphorylation and NADH, acting an electron donor for maintaining redox balance through its oxidation to NAD^+^. Although available nutrients are limited in the gut lumen, competing pathogens exploit them to support fermentative growth in the anaerobic environment by generating ATP. Accumulating evidence from mice infection experiments reveals that Enterobacteriaceae including *E*. *coli* and *C*. *rodentium*, and also *Akkermansia muciniphila* and *Clostridioides difficile* utilize nutrients such as carbohydrates for advantageous fitness in the gut lumen.^201^ ^,^ ^217-223^ Similarly, *S*Tm can also exploit carbohydrates for advantageous colonization. Antibiotic treatment increases inducible nitric oxide synthase (iNOS)-dependent oxidization of galactose and glucose, generating galactarate and glucarate, which confer a fitness advantage on Enterobacteriaceae, including *S*Tm and *E*. *coli*.^224^ Furthermore, galactitol is a key determinant for promoting the competitive growth between *S*Tm and *E*. *coli*/*Klebsiella* spp. *K*. *michiganensis* and *K*. *oxytoca* can inhibit initial colonization of *S*Tm by competition for a nutrient, galactitol.^225^ ^,^ ^226^ Likewise, *E*. *coli* enhances CR against *S*Tm by competing for galactitol and galactose in a microbial context-dependent manner during the initial growth phase.^227^ ^,^ ^228^ Importantly, the exclusive utilization of a distinct carbon source, galactitol, confers a competitive colonization advantage among *S*Tm strains.^229^ Furthermore, this fuels the transfer of antibiotic resistance plasmids in the mouse gut.^229^ A recent comprehensive analysis has revealed the carbohydrate metabolic capacity of *S*Tm in the murine gut.^230^ D-fructose and D-mannose utilization pathways support a context-independent growth of *S*Tm in the murine gut, whereas D-galactose appears to play an important role in the context-dependent colonization in microbiota-perturbed mouse models.^230^ *N*-acetylglucosamine and hexuronates (*β*-glucuronide and D-galacturonate) are context-dependent nutrient sources for *S*Tm luminal growth.^230^

### Terminal electron acceptors

Energy production mainly governs microbiota composition, as bacteria generate adenosine triphosphate (ATP) to sustain and increase growth rates, thereby outcompeting other microbial community members.^231^ Efficient production of ATP is done through redox reactions. An electron donor, such as carbohydrate glucose, provides electrons to a terminal electron acceptor, such as molecular oxygen (O_2_) or nitrate (NO_3_^-^), via an array of redox reactions. These reactions yield the energy for bacterial growth, and therefore the choice and availability of terminal electron acceptor governs the competition advantage of enteric bacteria including *S*Tm.^232^ ^,^ ^233^ Thus, the intestinal inflammation during *S*Tm gut infection increases the availability of electron acceptors, which drives the pathogen luminal expansion through oxygen and alternative respirations.

### Oxygen (a terminal electron acceptor)

Oxygen provides the highest redox potential for generating ATP. Inflammation-dependent perturbation of gut microbiota alters the availability of terminal electron acceptors, including oxygen, in the gut lumen, which is the most important factor affecting the bacterial luminal colonization levels, along with nutrient utilization.^232^ Redox balance profoundly influences the availability of electron acceptors, and determines the mode of bacterial growth, namely aerobic or anaerobic. Obligate aerobic bacteria cannot thrive in the absence of oxygen, whereas facultatively anaerobic bacteria such as *E*. *coli*, *S*Tm, and other Enterobacteriaceae can growth in both oxygen-present and oxygen-limited environments, in which the pathogens shift the metabolic mode to anaerobiosis from aerobiosis. Namely, in oxygen-present environments, *S*Tm grow by aerobic respiration using oxygen as a terminal electron acceptor, whereas anaerobic metabolism such as nitrate respiration allows for growth in oxygen-limited environments.^234^ Therefore, the availability of terminal electron acceptors provides a competitive advantage for Enterobacteriaceae colonization in the mouse intestine.^235^ ^,^ ^236^ In homeostasis, oxygen is maintained at lower levels in the cecal and colonic lumen (~0.6%) than in the duodenum and ileum (approximately 6%).^237^ ^,^ ^238^ This difference in the luminal levels of oxygen is attributable to the oxygen-consuming activity of mitochondria in large intestinal IECs, which exert *β*-oxidation that consumes oxygen and oxidizes microbiota-derived butyrate, producing ATP.^239^ ^,^ ^240^ This results in restriction of oxygen diffusion to large intestinal lumen, thereby creating hypoxia at the large intestinal mucosal surface and driving the microbiota to anaerobic fermentation.^241^ On the other hand, depletion of butyrate-producing microbiota members such as Clostridia by infection-induced inflammation or antibiotic treatment reduces *β*-oxidation activity and shifts epithelial metabolism to lactate fermentation, resulting in diffusion of oxygen into the gut lumen and thereby elevating luminal oxygen levels.^95^ Furthermore, genes encoding oxygen respiratory pathway are overrepresented in the dysbiotic microbiome.^242^ Antibiotic treatment depletes butyrate-producing Clostridia and thereby reduces epithelial signaling through the intracellular butyrate sensor peroxisome proliferator-activated receptor gamma (PPAR-*γ*), which leads to promotion of aerobic *S*Tm expansion in the gut lumen by using cytochrome bd-II oxidase (CyxAB).^95^ ^,^ ^243^ Besides *S*Tm, other Enterobacteriaceae strains and Clostridia species utilize oxygen-dependent respiration for their competitive colonization. Locus of enterocyte effacement (LEE)-encoded T3SS-induced colonic crypt hyperplasia provides *C*. *rodentium* access to oxygen, resulting in advantageous fitness in the intestinal tract.^244^ *E*. *coli* utilizes microbiota-derived formate as an electron donor in conjunction with oxygen respiration, conferring competitive fitness in the inflamed gut.^242^ It is notable that oxygen-dependent respiration confers a competitive growth advantage to the pathogen in the gut lumen. Acquisition of protective Clostridia in neonates before weaning contributes to prevention of the growth of enteric pathogens such as *S*Tm in the gut early in life by consuming oxygen, and thereby restricting growth of anaerobes.^245^ Endogenous Enterobacteriaceae including *Proteus vulgaris*, *P*. *mirabilis*, *K*. *oxytoca*, *Enterobacter cloacae* and *E*. *coli* exert aerobic metabolism by using oxygen as an electron acceptor, which confers niche protection against *S*Tm.^246^

### Nitrate (a terminal electron acceptor)

Besides molecular oxygen, *S*Tm infection-induced inflammatory responses provide alternative anaerobic electron acceptors including nitrate, which has a higher redox potential for ATP generation. As mentioned above, recruited neutrophils and monocytes produce superoxide (O_2_^−^) in the gut lumen via NADPH oxidase 2 (NOX2). In parallel, nitric oxide (NO) is produced by IECs or immune effector cells such as neutrophils and monocytes via iNOS. Thus, neutrophil-derived nitric oxide is converted to nitrate (NO_3_^-^) via interaction with superoxide in the gut lumen, and nitrate is utilized by *S*Tm for luminal expansion. In addition, SopE-dependent iNOS expression generates NO, which reacts with ROS to form peroxynitrite (ONOO^-^). Peroxynitrite isomerizes to nitrate, thereby promoting *S*Tm outgrowth by serving as an electron acceptor through NarL and/or NarP-regulated nitrate reductase complexes (NarGHJI, NarZYWV, and NapFDAGHBC).^247^ ^,^ ^248^ Nitrate respiration is linked to Tsr-mediated energy taxis, providing a fitness advantage in the inflamed gut.^202^ Importantly, aerobic respiration synergizes with nitrate respiration, and together these pathways drive not only luminal expansion but also fecal–oral transmission of *S*Tm.^95^ ^,^ ^243^ Collectively, cytochrome oxidases (CyxAB-encoded cytochrome bd-II oxidase and CydAB-encoded cytochrome bd oxidase) and nitrate reductase complexes (NarGHJI, NarZYWV, and NapFDAGHBC) play central roles in luminal expansion of *S*Tm in the inflamed gut.

### Tetrathionate (a terminal electron acceptor)

Hydrogen sulfide (H_2_S) is a metabolite produced by the gut microbiota, and IECs subsequently detoxicate it into thiosulphate (S_2_O_3_^2^^−^).^249^ ^,^ ^250^ During the intestinal inflammation, thiosulphate is converted to tetrathionate (S_4_O_6_^2^^−^) by neutrophil-derived oxidative burst.^51^ Thus, *S*Tm-induced gut inflammation generates a respiratory electron acceptor, tetrathionate, which is exploited by TtrSR-regulated TtrBCA-encoded tetrathionate reductase complexes, conferring a growth advantage on *S*Tm in the inflamed gut.^51^

### Fumarate (a terminal electron acceptor)

Fumarate respiration is a critical mode of energy conservation for bacterial growth under anaerobic conditions.^251^ ^,^ ^252^ *E*. *coli* uses fumarate as a terminal electron acceptor in the intestine by passing electrons to cytochrome bd oxidase and the anaerobic terminal reductases in the mouse intestine.^236^ C_4_-dicarboxylates (C4-DCs) such as L-aspartate and L-malate play an important role in the colonization of mammalian intestine by *E*. *coli*, *S*Tm, and other enteropathogenic bacteria.^253^ As mentioned above, DcuABC-dependent uptake of aspartate and malate and subsequent fumarate respiration—using either endogenously produced fumarate from hexose fermentation or exogenously supplied C4-DCs, promote initial growth in the gut.^76^ Moreover, beyond their role in the nonperturbed gut, DcuABC transporters support *S*Tm colonization in the inflamed gut lumen via fumarate reductase (Frd).^254^ Thus, C4-DCs play a central role in promoting gut colonization of *S*Tm, and further act as signaling molecules for adaptation to the host physiological environment.

### Fructose-asparagine, F-Asn (an electron donor)

F-Asn is an Amadori compound that is present at high concentrations in fruits, vegetables, and other foods.^255^ ^,^ ^256^ *S*Tm consumes F-Asn as a carbon source in concert with tetrathionate respiration during intestinal inflammation, leading to luminal expansion.^257^ In addition to *S*Tm, other Enterobacteriaceae members, such as *K*. *oxytoca*, *K*. *pneumoniae* and *C*. *rodentium*, and some strains of *Clostridium* can utilize F-Asn, suggesting that the *Clostridium* spp. likely compete with *S*Tm for F-Asn, leading to competitive exclusion of the pathogen.^258^ Moreover, inhibition of the F-Asn utilization pathway could be a potential therapeutic target, in which a toxic metabolite accumulates in *S*Tm, resulting in the non-growth of the pathogen.^258^ A recent work proposed combined administration of an inhibitor for F-Asn utilization and a probiotic as a new therapeutic intervention for *S*Tm gastrointestinal infection.^259^

### *S*- and *N*-oxides (an electron donor)

Interaction of the inflammation-derived ROS with organic sulfides forms reactive *S*-oxides, which act as terminal electron acceptors for *dmsABC*-encoded dimethyl sulfoxide (DMSO) reductases (DmsABC) of *S*Tm. DmsABC-mediated DMSO respiration contributes to *S*Tm colonization of the unperturbed anaerobic gut.^260^ Similarly, the ROS oxidize trimethylamine, yielding trimethylamine *N*-oxide (TMAO), which is a terminal electron acceptor for TMAO reductases (TorCAD). In chickens, colonization of the *Salmonella enterica* serovar Gallinarum is required for both DMSO and TMAO respirations.^261^

### Lactate (an electron donor)

The intestinal inflammation along with depletion of commensal Clostridia reduces epithelial oxygen consumption by decreasing mitochondrial activity in IECs.^262^ In IECs, glucose catabolism through aerobic glycolysis (fermentation) thus increases the luminal concentration of epithelium-derived L-lactate.^263^ Subsequently, *S*Tm uses the host-derived L-lactate as a nutrient acting as an electron donor, where oxygen is the terminal electron acceptor.^263^ Dysbiotic inflammation promotes anaerobic glycolysis, producing L-lactate from glucose, where *S*Tm utilize L-lactate for outgrowth in the inflamed gut.^243^ Furthermore, lactate fermentation by lactate dehydrogenase (LdhA) is more important than respiration using oxygen or alternative electron acceptors such as nitrate for colonization of the chicken ceca by *S*Tm.^264^ Notably, cholera toxin modulates the metabolism of host cells, resulting in an iron-depleted intestinal niche that allows for luminal expansion of *Vibrio cholerae* through acquisition of nutrients including L-lactate.^265^ In addition to serving as electron donors, host-derived oxygen and L-lactate provide cues for *S*Tm to regulate lactate oxidation metabolism on a transcriptional level, conferring a competitive advantage.^266^ Therefore, L-lactate can serve not only as an electron donor during fermentation but also as a critical cue that alters the transcriptional program, thereby enabling *S*Tm outgrowth in the inflamed gut.

### Ethanolamine (an electron donor)

Ethanolamine is a microbiota-derived fermentation product. Furthermore, ethanolamine is released into the intestinal lumen as a result of phospholipase D-mediated hydrolysis of endogenous phosphatidylethanolamine.^267^ ^,^ ^268^ In the inflamed gut, tetrathionate respiration enables *S*Tm that encodes the *eut* genes to use ethanolamine, a nutrient derived from phosphatidylethanolamine, for luminal growth.^269^ Thus, intestinal inflammation allows *S*Tm to consume specific nutrients via anaerobic respiration rather than fermentation. Ethanolamine utilization is prevalent in pathogenic *E*. *coli* and pathobionts and supports their growth in the intestinal tract.^270^ ^,^ ^271^ Thus, ethanolamine is an important nutrient for Enterobacteriaceae luminal growth in the gut, especially during inflammation.

### 1,2-Propanediol (an electron donor)

In addition to ethanolamine, 1,2-propanediol is a fermentation product. The *pdu* gene-encoded proteins of *S*Tm allow for consumption of 1,2-propanediol. The 1,2-propanediol utilization genes are expressed by the intestinal metabolic adaptation in *S*Tm and AIEC.^210^ ^,^ ^272^ ^,^ ^273^ Indeed, *S*Tm can consume a microbiota-derived fermentation product, 1,2-propanediol, for luminal growth in the mouse intestinal tract.^274^ On the other hand, tetrathionate respiration is coupled to ethanolamine utilization,^269^ whereas 1,2-propanediol utilization is required for both aerobic and anaerobic respiration,^274^ suggesting that *S*Tm can properly use the fermentation products.

### Formate (an electron donor)

Formate is a microbiota-derived metabolite that is utilized as an electron donor by *S*Tm *in vitro.* ^275^ During dysbiosis, formate concentrations in the gut are elevated. Enterobacteriaceae including *E*. *coli* and *C*. *rodentium* thus utilize microbiota-derived formate as an electron donor in conjunction with oxygen respiration, conferring competitive fitness in the inflamed gut.^242^ ^,^ ^244^ Notably, IEC death-induced nutrient release impacts a core transcriptional response of *S*Tm and acts as a source of fuel for the pathogen. Thus, pyruvate-lyase-encoding *pflB* gene is a key driver of the colonization, in which PflB produces pyruvic acid and formate under carbon limited condition.^276^ Furthermore, both formate production, which is involved in induction of T3SS-1 gene expression, and formate degradation play critical roles in *S*Tm colonization, especially within the mucus layer.^277^ ^,^ ^278^

### Succinate (an electron donor)

Succinate is an intermediate metabolite in the fermentation of indigestible dietary and host-derived carbohydrates into SCFAs or end-products by microbiota members including Bacteroidaceae, Prevotellaceae, and Clostridaceae.^279-281^ Certain resident bacteria utilize succinate as an energy source, instead of using carbohydrates. In mice, succinate supplementation in drinking water promotes *Clostridia* spp., probably due to consumption of oxygen by succinate utilization, leading to enhanced colonization of strict anaerobes.^245^ *C*. *difficile* utilizes microbiota-derived succinate by inducing a pathway that metabolizes it, allowing for efficient expansion and causing disease after antibiotic treatment or motility disturbance.^282^ Furthermore, succinate functions as a signaling molecule in pathogen–host interactions and as a nutrient for host cells, which is involved in intestinal disorders.^253^

Tricarboxylic acid (TCA) cycle, a central metabolic pathway, is necessary for luminal growth.^254^ In the unperturbed gut, the TCA cycle of *S*Tm is branched, with fumarate being reduced to succinate via fumarate reductase (Frd).^254^ ^,^ ^283^ Upon intestinal inflammation, the TCA cycle of *S*Tm is more oxidative due to the availability of electron acceptors such as nitrate, and succinate is converted to fumarate by succinate dehydrogenase (Sdh).^283^ Thus, the metabolic adaptation of *S*Tm to the inflamed gut provides alternative nutrient utilization that supports further luminal colonization. During initial growth, *S*Tm utilizes mixed-acid fermentation of hexose, such as acetate fermentation, and fumarate respiration producing succinate.^254^ Once gut inflammation occurs, *S*Tm adapts by starting ethanol fermentation for redox balancing and by supplying the oxidized TCA cycle with *α*-ketoglutarate for additional energy because hexoses appear to become limiting, and gluconeogenesis may be increasingly needed.^254^ Thus, the succinate-related mixed-acid fermentation plays a critical role in adaptation and growth of *S*Tm in the host.

### Resistance to inflammatory responses

Plasma B cells in Peyer’s patches and mLN contribute to IgA production in response to the commensal microbiota and SCFA, which shape the microbiota composition and diversity, thereby playing a critical role in gut homeostasis.^284^ ^,^ ^285^ *S*Tm invasion into the gut elicits production of the O-antigen specific IgA that controls gut luminal growth of the pathogen by promoting enchained growth.^52^ ^,^ ^286^ Furthermore, *S*Tm infection-induced inflammation also triggers the secretion of certain antimicrobials into the gut lumen, including lipocalin-2, calprotectin and RegIII C-type lectins such as RegIIIβ and RegIIIγ.^199^ ^,^ ^287-289^

Immune effectors (antimicrobial proteins, AMPs) are inducibly secreted into the gut lumen in response to *S*Tm infection, followed by intestinal inflammation. In the lamina propria during *S*Tm infection, conventional DCs (cDCs) release IL-23 by recognizing flagellin, which promotes IL-22 release by innate lymphoid cells (ILCs). The IL-22-dependent inflammatory responses trigger the production of AMPs, including lipocalin-2, RegIII lectin (RegIIIβ and RegIIIγ), and calprotectin by IECs and/or neutrophils, where the AMPs express antimicrobial activities by either directly killing pathogens or limiting the nutrient availability to pathogens in the gut lumen.^199^ ^,^ ^287-290^ Thus, resistance of *S*Tm to the inflammatory responses supports gut colonization in such adverse conditions.

### Lipocalin-2

Lipocalin-2 interferes with the acquisition of iron, an essential nutrient for bacterial growth, by sequestering a siderophore called enterochelin that allows for iron acquisition for enterobacteria, and thereby limits iron availability.^291^ The *iroBCDE iroN* gene cluster-encoded salmochelin, a glycosylated derivative of enterochelin, confers lipocalin-2 resistance because this siderophore does not bind to lipocalin-2. Therefore, lipocalin-2 resistance conferred by salmochelin supports *S*Tm colonization in the inflamed gut, where lipocalin-2 expression is induced by IL-17 and IL-22.^50^ ^,^ ^288^ Interestingly, a commensal bacterium can impact the iron availability between *S*Tm and the host. *Bacteroides thetaiotaomicron* acquires luminal iron by XusB, the siderophore-binding lipoprotein, which shields salmochelin from lipocalin-2.^292^ ^,^ ^293^ Thus, *S*Tm utilizes XusB-bound salmochelin for iron acquisition.^293^

### RegIII lectins

The RegIII lectins, including RegIIIβ and RegIIIγ, are a family of antimicrobial proteins. RegIIIβ permeabilizes the outer membrane of Gram-negative bacteria by binding to lipid A, and RegIIIγ recognizes peptidoglycan on Gram-positive bacteria, and thereby kills the bacteria through osmotic lysis.^101^ ^,^ ^294-296^ RegIIIβ harbors bactericidal activity toward certain Gram-negative and Gram-positive bacteria, whereas RegIIIγ can kill Gram-positive bacteria, but not Gram-negative bacteria.^102^ The expression levels of RegIIIβ are very low under homeostasis; however, gastrointestinal infection with *S*Tm can induce the production of RegIIIβ in the gut lumen.^103^ ^,^ ^199^ Importantly, *S*Tm is resistant to killing by RegIIIβ both *ex vivo* and *in vivo*, probably due to its robust outer membrane barrier and LPS-mediated steric hindrance.^49^ ^,^ ^103^ ^,^ ^294^ In the inflamed gut during *S*Tm infection, RegIIIβ reduces luminal levels of the cardinal microbiota member *Bacteroides* spp. by its bactericidal activity.^49^ This leads to alteration of the metabolic profile in the gut lumen, in which luminal levels of vitamin B6 are reduced, and prolongation of *S*Tm gut colonization at the later stages of enteric infection.^49^ Importantly, supplementation with vitamin B6 facilitates *S*Tm clearance from the gut and ameliorates enteropathy, indicating that the RegIIIβ/vitamin B6 axis could be a therapeutic target for *S*Tm gastrointestinal infection. Furthermore, RegIIIβ has distinct roles across *S*Tm infectious stages: RegIIIβ directly facilitates epithelial invasion and gut colonization at early stages,^103^ and at later stages, the RegIIIβ-dependent metabolic shifts prolong *S*Tm colonization.^49^

### Calprotectin

Similar to lipocalin-2, calprotectin sequesters metal ions, including zinc and manganese.^287^ Since acquisition of metal ions is required for bacterial growth, overcoming any limitations to the acquisition contributes to colonization in the intestinal tract. During *S*Tm gut infection, calprotectin is induced by neutrophils and suppresses growth of microbiota via the sequestration of zinc and manganese. In contrast, *S*Tm overcomes calprotectin-mediated zinc and manganese chelation by expressing the high affinity zinc transporter ZnuABC and the manganese transporters SitA and MntH.^287^ ^,^ ^288^ ^,^ ^297^ Therefore, *S*Tm can escape from the calprotectin-mediated inflammatory responses that limit its colonization.

### Interference competition

The microbiota produces small molecules and metabolites that interfere with growth of other bacteria. SCFAs and bacteriocins (also called microcins) are well-known examples that prevent the luminal growth and colonization of closely related species.^298-300^ In addition to these antimicrobials, enteric bacteria can kill other bacteria in a contact-dependent manner via T6SSs.

### SCFAs

SCFAs, including acetate, propionate and butyrate, are the most abundant microbiota-derived metabolites and are absorbed by IECs.^301^ In addition to their significant effects on host cells, SCFAs directly influence *S*Tm colonization in the gut lumen. Microbiota-derived propionate limits luminal growth of *S*Tm by disrupting intracellular pH homeostasis.^94^ Thus, microbiota-derived SCFAs influence luminal growth and colonization of *S*Tm and other pathogens by contributing not only to intestinal homeostasis via altering IEC metabolism and regulating the mucosal immune barrier,^95^ ^,^ ^241^ ^,^ ^302-305^ but also by modulating pathogen homeostasis and virulence gene expression.^89^ ^,^ ^94^ ^,^ ^96^

### Bile acids

Bile acids released into the gut lumen act as detergents that solubilize fats. In addition to this fundamental role, bile acids also have potent antimicrobial properties, thereby contributing to host defense by killing some pathogens and pathobionts, as evidenced by findings that secondary bile acids in the gut are potent against *C*. *difficile*.^306^ However, bile acids can also kill commensal bacteria, whereas enteropathogenic bacteria and pathobionts, including *S*Tm, *C*. *rodentium*, and AIEC, are generally bile-resistant in the gut lumen.^78^ ^,^ ^307-309^ Therefore, *S*Tm can exploit luminal bile acids to compete with commensal bacteria, promoting luminal growth.^78^ ^,^ ^307^

### Bacteriocin (microcin)

Bacteriocin shapes the microbiota community by targeting competitors in the gut lumen.^299^ Additionally, bacteriocin can eliminate the enteropathogenic bacteria. In the case of *S*Tm, inflammation-induced *E*. *coli* 8178 antimicrobial microcin H47 toxin can specifically alter *S*Tm growth by potentiating the microcin uptake via ROS-mediated derepression of *iroN* expression.^310^ ^,^ ^311^

### T6SSs

T6SSs inject toxin-like compounds into the cytoplasm of target bacteria and thereby kill them.^312^ ^,^ ^313^ *S*Tm uses a T6SS to compete with commensal bacteria by direct killing.^314^ Likewise, commensal bacteria can also use T6SSs to limit pathogen colonization. *C*. *rodentium* T6SS contributes to pathogen colonization by specifically targeting *E*. *coli*, whereas commensal *E*. *coli* Mt1B1 also employs a T6SS to compete with this pathogen in the gut.^313^

### Microbiota-derived metabolites fueling the colonization of STm

Germ-free mice are more susceptible to *S*Tm infection compared to mice harboring a complex microbiota.^315^ Thus, the microbiota clearly contributes to protection from enteropathogenic bacterial infection through a wide array of mechanisms that prevent luminal growth and colonization of the pathogens. Microbiota-derived metabolites are key mediators of microbiota-mediated infection resistance. For example, SCFA-mediated protection is well known. Furthermore, *Bacteroides acidifaciens*-derived vitamin B6 inhibits prolonged colonization of *S*Tm and ameliorates enteropathy during remission stages.^49^ On the other hand, certain microbiota-derived metabolites enhance the virulence potential of enteropathogenic bacteria, resulting in luminal expansion of the pathogens and enhanced colonization.

### Indole

Indole is a microbiota-derived metabolite that is abundant in the intestinal lumen and acts as a signaling molecule. At physiological concentrations in the gut lumen, EHEC and *C*. *rodentium* sense luminal indole, resulting in decreased expression of virulence genes.^316^ On the other hand, although high concentrations of microbiota-produced indole are present in the lumen, the indole concentration decreases at the surface of IECs due to epithelial absorption. Notably, indole at lower concentrations allows IsrR, an orphan indole sensing regulator, to act as an activator of LEE genes.^317^ Likewise, since indole has been shown to repress the activities of T3SS-1 and the flagellum,^318^ future work will be needed to clarify whether an indole concentration gradient can affect *S*Tm gut colonization.

### Polyamine

Polyamines, such as putrescine, spermidine and spermine, are positively charged aliphatic molecules found in living organisms.^319^ Due to their chemical nature, polyamines can bind to anionic compounds such as nucleic acids and proteins,^320-322^ and thereby play roles in maintaining nucleic acid structure, tuning gene expression profiles, and contributing to cellular processes such as cell proliferation and differentiation, redox balance, and apoptosis. Almost all colonic polyamines are derived from production by the microbiota, because diet-derived polyamines are rapidly adsorbed in the small inestine.^323^ ^,^ ^324^ Spermidine in the intestinal tract has anti-inflammatory effects, with anti-inflammatory M2-like macrophages being induced by spermidine supplementation,^325^ consistent with recent reports using murine colitis models.^326-328^ In the context of gastrointestinal infection with enteropathogenic bacteria, however, spermidine supplementation is likely to have adverse effects on murine health. Intracellular levels of polyamines in both mammalian cells and bacteria are tightly regulated by synthesis and uptake of extracellular polyamines, thereby maintaining homeostasis. Notably, the polyamine homeostasis of *S*Tm depends on uptake rather than intracellular synthesis.^329^ Spermidine uptake is required for functional expression of T3SS-1, since spermidine enables *S*Tm to assemble the needle subunits of the secretion system machinery.^329^ Furthermore, spermidine supplementation in mice by oral gavage of *S*Tm facilitates luminal growth and colonization of the pathogen, which may result from increased expression of genes involved in flagellar motility and nitrate respiration.^330^ This is also true for the pathobiont AIEC, suggesting that higher concentrations of polyamines, including spermidine, are a risk factor for gastrointestinal infection with enteropathogenic bacteria.^330^ Thus, targeting polyamine uptake and regulating luminal polyamine levels could be promising strategies for developing therapeutic interventions for gut infection with enteropathogenic bacteria, including *S*Tm, and for AIEC-associated Crohn’s disease.

## Conclusion and future aspects

Mammalian hosts, including humans and mice, have sophisticated defense systems to prevent invasion by enteric pathogens, a phenomenon termed “colonization resistance” (CR) that is microbiota-dependent.^9^ In this framework, overlap in nutrient utilization profiles between commensal bacteria and pathogens is particularly important.^331^ Thus, to prevent and treat gastrointestinal infection, increasing CR through microbiota-targeted therapy may be useful. For instance, fecal microbiota transplantation (FMT) is recognized as an effective therapy for patients suffering from recurrent *C*. *difficile* infections (rCDI) by restoring the microbiota and thereby CR. In a similar context for *S*Tm gut infection, earlier work showed that a defined microbial community consisting of 33 bacterial strains isolated from human stool—a consortium originally developed to treat rCDI—confers protection against *S*Tm infection in a murine model, suggesting that complex microbial communities may be useful as therapeutic interventions for gastrointestinal infection caused by enteropathogenic bacteria, including *S*Tm.^332^ Furthermore, a recent study showed that a similar commensal bacterial consortium comprising 18 strains isolated from the stool of healthy humans (the F18-mix) has decolonization capacity against Enterobacteriaceae, including *K*. *pneumoniae* and *E*. *coli*, by regulating gluconate availability.^333^ On the other hand, we are still far from defining principles to predict which specific microbiota members can antagonize the pathogen infection by increasing CR activity. Currently, it is apparent that single species are generally insufficient for microbiota-based therapy. However, the same study also showed that, in the presence of the F18-mix, Enterobacteriaceae exhibit altered nutrient use, with *Klebsiella* preferentially consuming the favored carbon source gluconate.^333^ Furthermore, a recent study proposed “vaccine-enhanced competition,” which combines a whole-cell inactivated oral vaccine with a live niche competitor.^334^ Secreted IgA into the gut lumen promotes clearance of *S*Tm by “enchaining” pathogen cells, thereby imposing an evolutionary trade-off on IgA-targeted bacteria.^52^ ^,^ ^286^ ^,^ ^335^ Importantly, vaccine-mediated clearance from the gut lumen depends on metabolic niche overlap with competitors that can outcompete enteric pathogens. Thus, it is concluded that targeted therapeutic strategies such as microbiota-targeted therapies and vaccine-enhanced competition may act through a common principle: reinforcing selective immune responses (e.g., defined protective commensal bacterial member or IgA induced by vaccination) while also targeting molecules that support pathogen growth (e.g., gluconate) or introducing metabolically overlapping competitors. These approaches may therefore enable decolonization of Enterobacteriaceae, including *S*Tm, *Klebsiella*, and *E*. *coli*. Therefore, it will be important to identify pathogen-, host-, and microbiota-derived metabolites that support luminal growth and colonization of enteropathogenic bacteria.
